# GENETIC DELETION OF THE PRESINILIN‐LIKE PEPTIDASE SPPL2B SIGNIFICANTLY REDUCES NEUROINFLAMMATION AND HALTS PRODUCTION AND PLAQUE DEPOSITION IN PRE‐CLINICAL MODELS OF Alzheimer's DISEASE

**DOI:** 10.1002/alz.093828

**Published:** 2025-01-09

**Authors:** Jack Badman, Gefei Chen, Sandra Pérez Garcia, Per Nilsson, Simone Tambaro

**Affiliations:** ^1^ Karolinska Institutet, Solna Sweden; ^2^ Karolinska Institutet, Huddinge Sweden

## Abstract

**Background:**

Signal Peptide Peptidase‐Like 2b (SPPL2b) is relevant for AD, being a brain‐specific intramembrane protein involved in the cleavage of Alzheimer's disease (AD)‐related proteins, such as BRI2, inflammatory‐related proteins like CD74, TNFalpha, and Clec7a, and synaptic proteins Neuregulin‐1 and VAMP 1‐4. SPPL2b is specifically expressed in the hippocampus and cortex. The cleavage of TNFalpha by SPPL2b promotes the inflammatory pathway. Conversely, the SPPL2b substrate BRI2 regulates amyloid precursor protein (APP) cleavage and subsequent Aß production. This work explores the pathophysiological role of SPPL2b in AD pathogenesis and investigates the potential of inhibiting SPPL2b activity as a novel therapeutic strategy.

**Method:**

To characterize the role of SPPL2b in the APP cleavage process and neuroinflammation, we used neurons and glia primary cell cultures from WT and SPPL2b‐deficient mice. Furthermore, we assessed the therapeutic potential of inhibiting SPPL2b by employing a new AD mouse model generated through crossbreeding state‐of‐the‐art AppNL‐G‐F knock‐in mice with SPPL2b‐deficient mice. Cells and mouse brain samples were analyzed through western blotting, immunoprecipitation, and immunofluorescence. Aß levels were quantified using ELISA kits.

**Result:**

The results showed a significant increase in BRI2, followed by a substantial reduction in soluble APP, Aß40, and Aß42 levels in the conditioned media of SPPL2b KO neurons. Immunoprecipitation of BRI2 from the cortex of those mice revealed an increased interaction between BRI2 and APP in SPPL2b KO/AppNL‐G‐F mice. Most importantly, a notable decrease in brain Aß plaque deposition and a significant decrease in both cortical and hippocampal gliosis were observed at 4 months‐of‐age in SPPL2b KO/AppNL‐G‐F mice.

**Conclusion:**

The results outlined in this study support a relevant connection between SPPL2b and the development of Aß pathology in AD. In a global scenario characterized by the need to identify novel strategies to prevent and counteract AD progression, this study highlights and strengthens the importance of SPPL2b as a novel therapeutic approach for AD.

